# A two-stage classification approach identifies seven susceptibility genes for a simulated complex disease

**DOI:** 10.1186/1753-6561-1-s1-s30

**Published:** 2007-12-18

**Authors:** Nathan Pankratz

**Affiliations:** 1 Department of Medical and Molecular Genetics, 410 West 10th Street, HS4000, Indiana University, Indianapolis, Indiana 46202-3002, USA

## Abstract

The simulated data set of the Genetic Analysis Workshop 15 provided affection status, four quantitative traits, and a covariate. After studying the relationship between these variables, linkage analysis was undertaken. Analyses were performed in the first replicate only and without any prior knowledge of the underlying model. In addition to the main effect of the DR locus on chromosome 6, significant linkage was also identified on chromosomes 8, 9, 11, and 18. Notably, the power to detect linkage increased after transforming the skewed and kurtotic IgM and anti-CCP distributions. Moreover, genes on chromosome 11 could not be discerned from noise without the transformation, thus highlighting the need in real life situations for careful examination of the phenotypic data prior to genetic analysis. Significant association with one single-nucleotide polymorphism was identified for the regions on chromosome 11 and 18. Haplotype analyses were attempted for the other regions, but only the underlying variation of the DR locus could be identified. Two methods were then applied to predict classification using the factors identified so far. These methods – logistic regression and multifactor dimensionality reduction (MDR) – performed comparably for this data set. Those affected individuals that were misclassified as unaffected were then used in a genome-wide association analysis to identify additional susceptibility loci. Two additional loci were identified in this fashion, illustrating the usefulness of this two-stage classification approach.

## Background

Studies designed to identify genes contributing to complex diseases have been ongoing for many years, utilizing different study designs and methods with varied success. The simulated data set of the Genetic Analysis Workshop 15 (GAW15) provided an opportunity to evaluate different analysis techniques used to identify genes underlying complex phenotypes. I have also attempted to predict disease status given the presence of apparent "high risk" alleles (those alleles associated with disease or endophenotypes) identified in analyses, with the intent of further studying those individuals that are misclassified. Ideally, these individuals will be enriched for susceptibility genes not identified in the first round of analyses.

## Methods

All analyses were performed using the simulated data set without knowledge of the underlying model, including number of genes or their location. Analyses were performed with complete genotypic and phenotypic data from Replicate 1.

### Phenotype

The simulated data set included a number of continuous traits as well as affection status for the simulated disease. The alleles at the main susceptibility locus (DR) were also provided in the phenotype file, as was smoking history. The relationships between these variables were inferred using linear and logistic regression, and significant results were included in subsequent analyses as covariates.

### First-stage genetic analysis

A genome screen was performed for affection status using the nonparametric linkage analysis methods implemented in Mapmaker/SIBS [1]. Quantitative trait locus (QTL) analysis was also performed for age at onset, severity, IgM, and anti-CCP using both Haseman-Elston regression [2] and the variance components method implemented in SOLAR [3]. Those trait distributions with unacceptable levels of kurtosis were adjusted using a natural log transformation before being analyzed.

Association analyses were performed for SNPs contained within regions with a LOD score greater than 3.0, using the pedigree disequilibrium test (PDT) [4]. Continuous traits were analyzed with linear regression using additive models.

The provided (phased) haplotypes were used in windows increasing from one to six SNPs for each chromosomal region to determine if haplotypes were more powerful than individual SNPs at predicting any particular trait. *R*^2 ^values were maximized in this way, with the understanding that a parsimonious model was preferable given comparable *R*^2 ^estimates.

### Prediction of phenotype

Logistic regression and multifactor dimensionality reduction (MDR) [4] were used to quantify the effects of the apparent high-risk alleles identified in these genetic analyses. While the MDR software is designed to classify cases and controls, logistic regression only provides a predicted value between zero and one for each individual. Therefore, a threshold for these predicted values was chosen by maximizing sensitivity and specificity and was then used to dichotomize the predicted values into cases and controls. For both methods, the dependent variable was affection status, and the number of copies for each high-risk allele was used as an independent variable. Smoking history and gender were also included where appropriate. The first affected sibling from each family was used along with an equal number of controls. The same approach using linear regression was also used for the quantitative traits.

### Second-stage genetic analysis

Those affected individuals that were misclassified by the MDR algorithm were used in a genome-wide association study. PDT was used to ensure that trios from the same family would not bias the test statistic.

## Results

### Phenotype

Affection status was significantly associated with the DR locus (OR: 25.2 for each DR_3 allele, OR: 3.0 for each DR_2 allele), gender (OR: 2.8 for females), and smoking history (OR: 2.5 for previous smoker). The overall Cox & Snell *R*^2 ^for the model was 0.515, with a majority of the influence coming exclusively from the DR locus (Cox & Snell *R*^2 ^= 0.493).

IgM was significantly associated with smoking and, to a lesser extent, with gender, but not with the DR alleles. Anti-CCP was only significantly associated with the DR_3 allele. Severity was not associated with any covariate. Age at onset was significantly associated with the DR_3 allele, smoking history, and female gender.

### Genetic analyses

A genome screen of the qualitative phenotype (affected versus unaffected) yielded a significant LOD score only for the region on chromosome 6 harboring the DR locus. QTL analysis of the quantitative traits yielded significant LOD scores for a total of five chromosomal regions (see Table 1). IgM and anti-CCP were transformed to reduce the levels of kurtosis (from 33.4 to 0.6 and from -1.4 to 0.7, respectively).

**Table 1:** Genome screen results (LOD scores) for replicate 1

	Chromosome [Location (cM)]				
	
Trait (any covariates included)	6 (49 cM)	8 (169 cM)	9 (50 cM)	11 (115 cM)	18 (94 cM)
Affection status					
Mapmaker/SIBS	50.4				
AgeAtOnset (including gender)					
Haseman-Elston		3.1	4.4		
SOLAR		4.2	4.5		
Severity					
Haseman-Elston		8.6	9.6		
SOLAR		9.0	7.5		
Anti-CCP† (including gender)					
Haseman-Elston	5.4				(1.8)
SOLAR	4.9†				(2.2)
Anti-CCP† (including gender and DR alleles)					
Haseman-Elston	(1.0)				3.3
SOLAR	(1.3)				3.8†
Anti-CCP transformed (including covariates)					
Haseman-Elston	(0.5)				5.1
SOLAR	(1.2)				7.9
IgM†(including gender and smoking history)					
Haseman-Elston				(1.2)	
SOLAR				4.9†	
IgM transformed (including covariates)					
Haseman-Elston				10.8	
SOLAR				22.4	

†Results from SOLAR for these untransformed variables should not be considered valid because these trait distributions had high levels of kurtosis and were the only ones to yield a lot of noise (additional findings on other chromosomes with LOD scores approaching and exceeding 3.0 that are assumed to be false positives).

PDT analysis of SNP data from each of the affected families yielded significance on chromosome 6 near the DR locus, as well as on chromosomes 11 (SNP11_387 and SNP11_389; *p *= 10^-6 ^and *p *= 6 × 10^-16^) and on chromosome 18 (SNP18_269; 5 × 10^-6^). A Bonferonni correction for 8723 tests yields an alpha of 5 × 10^-6^. All other SNPs had *p*-values below this threshold and were therefore not significant.

Linear regression identified several SNPs that contributed to the variation in the quantitative traits. Those SNPs were then used to form haplotypes of varying sizes in an attempt to characterize their influence. SNP11_389 alone captured nearly 50% of the variance within the transformed distribution of the IgM trait, after accounting for the effects of gender and smoking, a result that did not improve with a larger haplotype. This SNP was also a significant predictor of affection status (*p *= 10^-32^).

The deleterious haplotype for the region on chromosome 18 was not obvious; however, the haplotypes resulting from SNP18_268, SNP18_269, and SNP18_270 did the best at predicting anti-CCP levels (*p *= 10^-16^). SNP18_269 by itself, however, was best at predicting affection status (*p *= 2 × 10^-8^).

Determining the specific deleterious haplotype for the region on chromosome 9 was not straightforward. The best four SNPs appear to be SNP9_185, SNP9_186, SNP9_189, and SNP9_190. When predicting severity, the *R*^2 ^value was fairly low (*R*^2 ^= 0.040; *p *= 5 × 10^-9^) given the size of this haplotype. The selected SNPs were not at all associated with affection status (*p *= 0.391). Similarly, no association with either severity or affection status could be identified for any of the SNPs under the chromosome 8 linkage peak.

### Prediction of phenotype

After adding in the newly identified deleterious SNPs (SNP11_389 and SNP18_269) to the model described above, the Cox & Snell *R*^2 ^increased to 0.531 (The Nagelkerke *R*^2 ^increased from 0.683 to 0.705). The addition of these two factors improved the results from MDR from a balanced testing accuracy of 85.7% to 86.5% (See Table 2 for a summary). All individuals with two DR alleles were classified as affected by the final model. Those with only one DR allele were classified based upon other factors. No individuals without a DR allele were classified as affected. Those 405 affected individuals who were misclassified as unaffected by the MDR algorithm were then used in the second stage of the analysis.

**Table 2:** Classification results

	Logistic regression		
		
MDR testing accuracy	Maximized accuracy	Nagelkerke *R*^2^	Factors
*Before undertaking genetic analyses*			
82.6%	84.3%	0.652	Number of DR 3 alleles
85.7%	85.7%	0.683	Number of DR 3 alleles, sex, smoked
*Using results from first round of genetic analyses*			
86.5%	86.4%	0.705	Number of DR 3 alleles, sex, smoked, SNP11_389, SNP18_269
*Using results from second round of genetic analyses*			
88.4%	88.5%	0.715	Number of DR 3 alleles, sex, SNP6_154, SNP6_162
88.2%	88.5%	0.746	+Smoked, SNP11_389, SNP18_269
*Using SNPs/SNP haplotypes nearest the underlying loci as described in the "answers"*			
88.5%	88.5%	0.746	The SNPs nearest loci A-F, sex, smoking, DR genotype
*Using the underlying loci provided in the "answers"*			
88.7%	90.1%	0.773	Number of deleterious alleles at loci A-F, sex, smoking, DR

### Second-stage genetic analysis

Two loci, SNP6_154 (*p *= 5.2 × 10^-9^) and SNP6_162 (*p *= 1.6 × 10^-6^), exceeded a Bonferroni correction for 9187 tests (α = 5.6 × 10^-6^).

## Discussion

**Figure 1:**
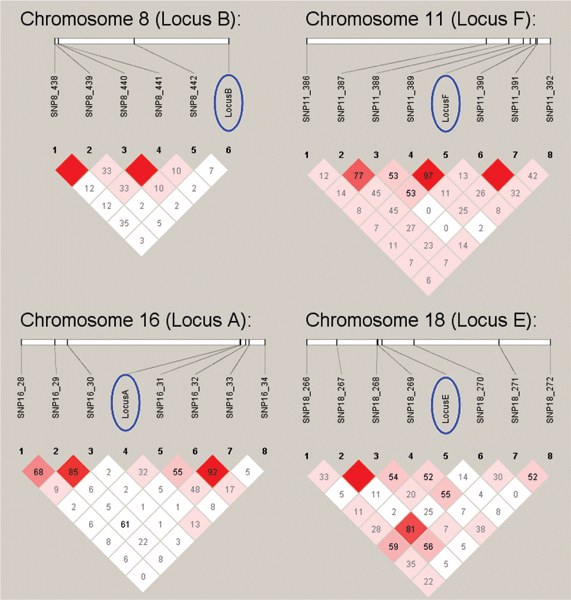
**LD between underlying loci and surrounding SNPs**. The moderate to strong LD between locus E, locus F, and their respective nearest markers explains why these loci were easy to detect. Similarly, the lack of LD (*r*^2 ^= 0.0) between locus A, locus B, and their respective nearby markers explains why association with these loci could not be detected.

Without prior knowledge of the underlying model, this two-stage, classification strategy identified linkage to all eight loci except for what was referred to as locus A and identified significant association to all loci except for locus A and B. When linkage disequilibrium (LD) was assessed between these two underlying SNPs and the SNPs nearest to them in the provided data set, it becomes clear that the reason for the false negatives was the absence of LD (*r*^2 ^= 0.00). Those SNPs that could be identified were in LD with their nearest marker (See Figure 1).

The level of kurtosis in the IgM trait was an unacceptable 33.4, but transforming the trait distribution not only brought the kurtosis (and therefore the false positive rate in SOLAR) under control, but it increased its power to detect linkage as well. Moreover, locus F on chromosome 11 could not be discerned from noise without this transformation. While the kurtosis of anti-CCP started off at -1.4, its power to detect linkage was also improved through transformation.

**Figure 2:**
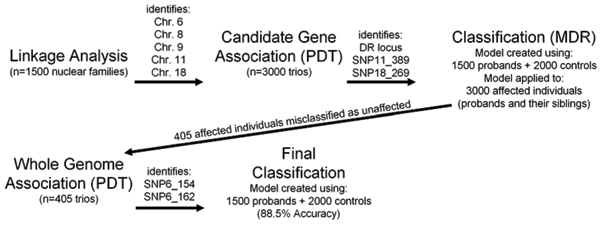
Summary of methods and samples for this two-stage classification strategy.

Prediction accuracy improved from 86% at baseline to 89% after two rounds of genetic analyses. Upon receiving the "answers" (a description of the underlying model), the classification methods were rerun using the markers directly surrounding the underlying causal loci and by directly modelling the mode of inheritance for each locus. This posterior information did not improve the prediction accuracy (Table 2). When the underlying genotypes of the susceptibility loci that were provided with the Answers were analyzed, classification accuracy only improved to 90% (Table 2, Figure 2).

## Conclusion

Incorporating information gained from linkage analysis and candidate gene association studies can create a subsample that is enriched for previously unidentified genes and thereby improves power to detect these additional loci in a whole-genome association analysis. Two loci (C and D) that were previously "over shadowed" by the DR locus were identified by factoring out information gained in the first round of analyses. While this study used linkage and candidate gene information, the source of prior information could also have come from another source, such as a previous whole-genome association (particularly one using a different type of sample, for instance cases and controls instead of trios). Similarly, other classification methods could be use instead of regression or MDR.

For these data, MDR did not outperform logistic regression. This is most likely due to the underlying simulation parameters. Since the only gene × gene or gene × environment interactions modelled were impossible to detect (*r*^2 ^= 0.0 for all markers surrounding Locus A and Locus B), there were no high order interactions for MDR to identify – the software's raison d'etre. Similarly, the simulation parameters assumed the common disease, common gene hypothesis in which the same high-risk allele either arose several times independently or arose so long ago that the background haplotype was almost completely degraded. Both situations would mean that haplotype methods, particularly for the marker density provided, could not provide more information than the analysis of single SNPs independently.

An additional consequence of the underlying simulation parameters resulted from its design to mimic an existing rheumatoid arthritis data set, which appears to contain a DRB1 allele with a large effect. In the simulated data set, this large effect realistically masked the signal of two smaller genes that were rendered undetectable if the effect of the larger gene was not taken into account. The two-stage classification approach used in this paper is one way to address this issue. A strategy such as this may prove to be equally successful in a real data set.

## Competing interests

The author(s) declare that they have no competing interests.
